# Interpreting the clinical significance of multiple large-scale mitochondrial DNA deletions (MLSMD) in skeletal muscle tissue in the diagnostic evaluation of primary mitochondrial disease

**DOI:** 10.3389/fphar.2025.1507493

**Published:** 2025-04-09

**Authors:** Jing Wang, James T. Peterson, Joaquim Diego D. Santos, Ada J. S. Chan, Maria Alejandra Diaz-Miranda, Imon Rahaman, Jean Flickinger, Amy Goldstein, Emily Bogush, Elizabeth M. McCormick, Colleen C. Muraresku, Vernon E. Anderson, Matthew C. Dulik, Douglas C. Wallace, Rui Xiao, Marni J. Falk, Angela N. Viaene, Zarazuela Zolkipli-Cunningham

**Affiliations:** ^1^ Division of Genomic Diagnostics, Department of Pathology and Laboratory Medicine, The Children’s Hospital of Philadelphia, Philadelphia, PA, United States; ^2^ Department of Pathology and Laboratory Medicine, Perelman School of Medicine, University of Pennsylvania, Philadelphia, PA, United States; ^3^ Mitochondrial Medicine Frontier Program, Division of Human Genetics, Department of Pediatrics, The Children’s Hospital of Philadelphia, Philadelphia, PA, United States; ^4^ Department of Pediatrics, Perelman School of Medicine, University of Pennsylvania, Philadelphia, PA, United States; ^5^ Center for Mitochondrial and Epigenomic Medicine, Division of Human Genetics, Department of Pediatrics, The Children’s Hospital of Philadelphia, Philadelphia, PA, United States; ^6^ Department of Biostatistics, Epidemiology and Informatics, University of Pennsylvania Perelman School of Medicine, Philadelphia, PA, United States; ^7^ Department of Pathology and Laboratory Medicine, Children’s Hospital of Philadelphia, Philadelphia, PA, United States

**Keywords:** primary mitochondrial disease (PMD), mitochondrial DNA (mtDNA), electron transport chain (ETC) enzymatic activity, ragged red fibers (RRF), ragged blue fibers (RBF), multiple large-scale mitochondrial DNA deletions (MLSMD)

## Abstract

**Background and Objectives:**

Improved detection sensitivity from combined Long-Range PCR (LR-PCR), Next-Generation Sequencing (NGS), and droplet digital PCR (ddPCR) to identify multiple large-scale mtDNA deletions (MLSMD) and quantify deletion heteroplasmy have introduced clinical interpretation challenges. We sought to evaluate clinical, biochemical, and histopathological phenotypes of a large clinical cohort harboring MLSMD in muscle to better understand their significance across a range of clinical phenotypes.

**Methods:**

A single-site retrospective study was performed of 212 diagnostic muscle biopsies obtained from patients referred for Primary Mitochondrial Disease (PMD) evaluation with muscle mitochondrial (mt)DNA sequencing performed at our institution, including electronic medical record (EMR) review of symptoms, biochemical results, and Mitochondrial Myopathy Composite Assessment Tool (MM-COAST) scores.

**Results:**

MLSMD were identified in 50 of 212 (24%) diagnostic tissue biopsies, and were universally present. in subjects ≥50 years (n = 18/18). In 45 of 50 (90%) subjects with MLSMD, no definitive genetic etiology was identified, despite clinical whole exome sequencing (WES) and/or whole genome sequencing (WGS). MLSMD heteroplasmy levels quantified by ddPCR ranged from 0% to 33%, exceeding 10% heteroplasmy in 5/45 (11%). Subjects with MLSMD (n = 45) were more likely to demonstrate mitochondrial abnormalities on histopathology, upregulation (≥150% of control mean) of one or more electron transport chain (ETC) complex enzyme activities, and reduced citrate synthase indicative of mitochondrial depletion (<60% of control mean) relative to subjects without MLSMD (n = 155). As clinical phenotypes varied across the MLSMD cohort, Bernier diagnostic criteria major/minor symptoms were used to discriminate 13 of 45 subjects with “suspected” PMD having unrevealing WES/WGS results and 32 of 45 subjects scored as “less likely” to have PMD. Relative to the “less likely” cohort, a significantly higher frequency of biochemical and muscle histopathological abnormalities (ragged red and COX negative fibers) were observed in the “suspected” cohort, further supporting a higher index of suspicion for PMD, p < 0.05.

**Discussion:**

MLSMD in skeletal muscle tissue were a common molecular finding (24%) in our cohort and consistently present in subjects ≥50 years. Among those with genetically undiagnosed MLSMD (n = 45), the “suspected” PMD subset (n = 13/45) represent a promising cohort for novel gene discoveries.

## 1 Introduction

Multiple large-scale mitochondrial DNA (mtDNA) deletions (MLSMD) refer to large-scale mtDNA deletions of different sizes co-occurring in a single specimen (Carrozzo et al., 1998; Copeland, 2008). MLSMD are typically identified in post-mitotic tissues, such as skeletal muscle tissue (Carrozzo et al., 1998; Copeland, 2008). MLSMD are known to be associated with nuclear genetic defects involved in mtDNA replication, repair, and maintenance, as well as mitochondrial dynamics (Copeland, 2008; Ahmed et al., 2015; El-Hattab et al., 2018). In general, low heteroplasmy levels of MLSMD can be tolerated at the tissue or cellular level because large numbers of intact mtDNA molecules remain, with compensatory effect to maintain normal muscle oxidative phosphorylation (OXPHOS) function (Campbell et al., 2014).

The first-line diagnostic testing in primary mitochondrial disease (PMD) is non-invasive molecular testing, aimed at identifying pathogenic variants in nuclear DNA (nDNA) and mitochondrial (mt)DNA. A diagnostic muscle biopsy facilitates mtDNA sequencing in muscle tissue, as well as mitochondrial functional assessment by electron transport chain (ETC) enzymatic activity, mtDNA content quantitation, and histopathological studies (Hinojosa and Bhai, 2023). Single amplicon long range PCR (LR-PCR) and Next-Generation Sequencing (NGS) based mtDNA testing have vastly enhanced the detection sensitivity for MLSMD in skeletal muscle tissue, leading to the ability to identify MLSMD down to <10% heteroplasmy level (Wang et al., 2022). The improved detection sensitivity of identifying MLSMD introduced clinical interpretation challenges as the clinical significance of MLSMD is not always clear. Indeed, in some cases, MLSMD may be related to the individual’s underlying genetic etiology or related to the normal aging process (Seidman et al., 1997; Lujan et al., 2020; Kraytsberg et al., 2006; Larsson, 2010). There are broad differential diagnoses associated with MLSMD that include non-PMD genetic etiologies that may present with a progressive multisystem disorder (Chinnery, 1993), neurodegenerative diseases (Campbell et al., 2011; Gu et al., 2002; Keogh and Chinnery, 2015), and non-genetic etiologies including environmental, alcohol and drug exposures (Payne et al., 2015; Mansouri et al., 1997; Tsuchishima et al., 2000), which highlight the clinical interpretation challenge of MLSMD in the evaluation of PMD. We sought to characterize the clinical, biochemical, and histopathological characteristics of subjects identified with MLSMD in skeletal muscle tissue evaluated in our Mitochondrial Medicine Frontier Program (MMFP) at the Children’s Hospital of Philadelphia (CHOP). We hypothesized that subjects with PMD-related MLSMD in muscle would be clinically distinct as compared to those subjects less likely to have a PMD genetic etiology. This is the first study to report the varying clinical, biochemical, and histopathologic phenotypes across a large cohort of subjects identified with MLSMD through diagnostic muscle biopsies, in whom a genetic etiology of PMD was not identified on clinical whole exome and/or whole genome sequencing.

## 2 Methods

### 2.1 Subjects and samples

**FIGURE 1:**
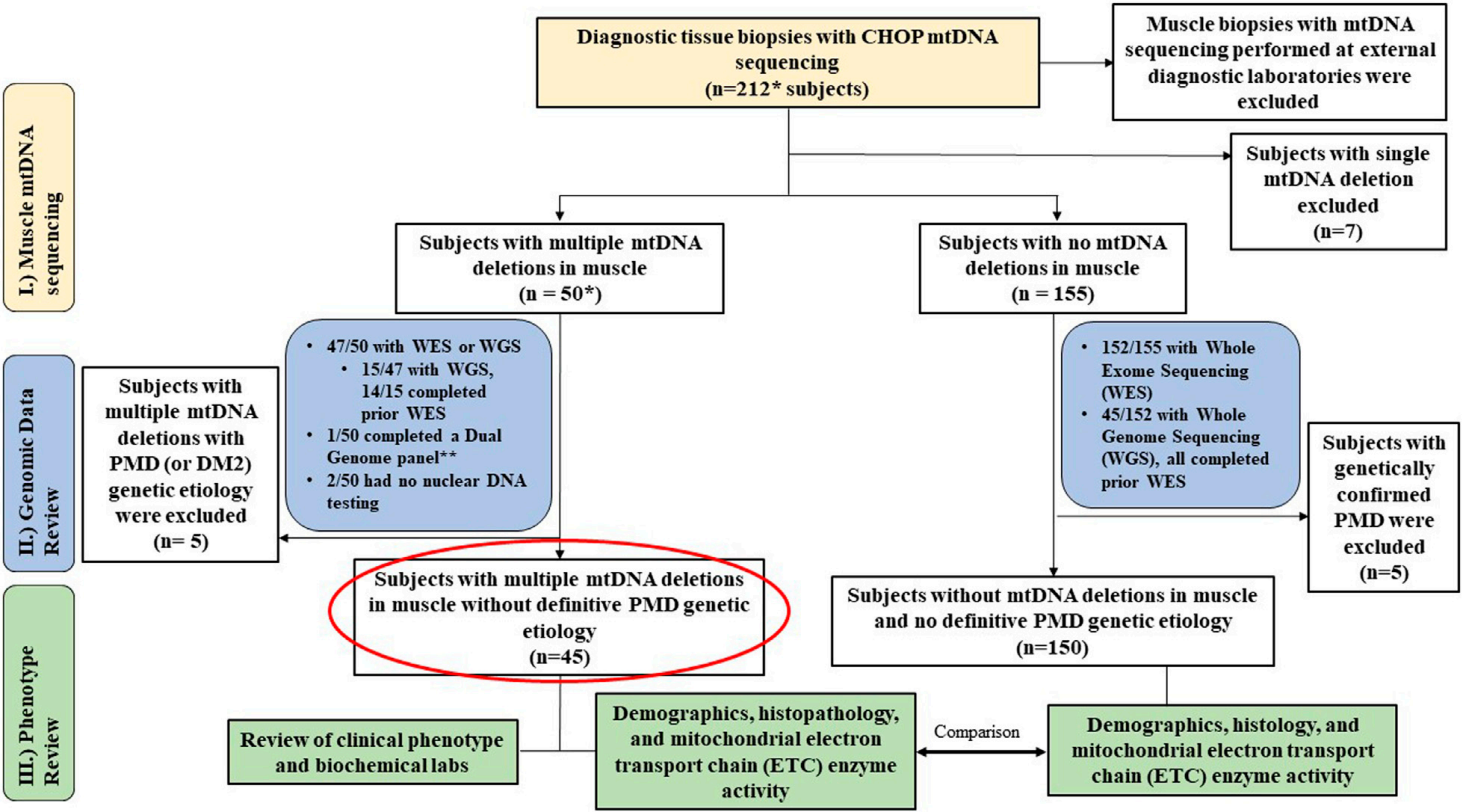
Study Overview. Flowchart outlines the study cohort and analyses conducted. * Includes 1 cardiac biopsy case. ** resulted in m.3243A>G considered diagnostic (14% in blood, 42% in muscle). MLSMD = multiple large-scale mitochondrial DNA deletions. PMD = Primary mitochondrial disease. DM2 = myotonic dystrophy type 2.

This was a single-center study involving retrospective chart review of diagnostic muscle biopsies performed in subjects previously referred for a PMD evaluation. All enrolled subjects had mtDNA sequencing and deletion analysis performed in muscle tissue (CHOP MitoGenome Test, Figure 1, https://www.chop.edu/centers-programs/mitochondrial-medicine-program/advanced-testing-mitochondrial-disease). A total of 212 diagnostic open muscle biopsies obtained under general anesthesia, comprising 211 vastus lateralis muscle biopsies and 1 cardiac muscle biopsy obtained between 2018–2023 were included for analyses under Institutional Review Board (IRB) approved study protocols (#23-020790, Wang, PI and #08-006177, Falk, PI). All muscle tissue specimens were flash frozen at collection in either isopentane for histology and/or in liquid nitrogen for all other diagnostic testing, then stored in a −70°C freezer until clinical diagnostic testing was performed. The study protocol included the requirement to review the CHOP MitoGenome raw data (JW) in each subject for accuracy. Diagnostic muscle biopsies with muscle mtDNA sequencing performed at an external diagnostic laboratory were excluded from analysis (Figure 1) as i) external results precluded review of raw mitogenome data and ii) varying diagnostic laboratory protocols prohibited a direct comparison of muscle mtDNA sequencing results.

### 2.2 MitoGenome test and MLSMD heteroplasmy quantification

Total nuclear and mitochondrial DNA was extracted using a commercially available DNA isolation kit (Gentra Systems Inc., Minneapolis, MN, United States). The entire mtDNA genome was amplified as a single linear amplicon using a high-fidelity long-range PCR (LR-PCR) system (PlatinumTM SuperFiTM Master Mix, ThermoFisher Scientific), followed by NGS with an average sequencing depth of 20,000x. Comprehensive mtDNA analyses were performed to detect mtDNA single nucleotide variations (SNVs) and large-scale single and multiple mtDNA deletions by using an in-house custom-built bioinformatics pipeline (Wang et al., 2022). When large-scale mtDNA deletions were detected, the heteroplasmy level of large-scale mtDNA deletions was quantified by a droplet digital PCR (ddPCR)-based assay. Two ddPCR reactions were carried out: one targeted the mtDNA commonly deleted region (*MT-ND4*), and the other targeted a region that is rarely involved in mtDNA large deletions (*MT-RNR2*) (Supplementary Figure S1A). These two ddPCR reactions were used to determine the number of non-deleted mtDNA molecules and total mtDNA molecules, respectively. The deletion heteroplasmy level was calculated as $\left[1-\left(\frac{ND4}{RNR}\right)\right]*100\%$ . To establish the lower limit of heteroplasmy detection using our assay, we performed serial dilution of a sample with a previously determined mtDNA deletion heteroplasmy, utilizing a DNA sample without mtDNA deletions from age and tissue typed matched control as a diluent. Results revealed that our ddPCR method reliably detects mtDNA deletions in samples with 10% or higher heteroplasmy levels. However, ddPCR is less reliable at detection of heteroplasmy levels <10% or in cases where the results overlap with those of normal controls (Supplementary Figure S1B). The ddPCR assay was repeated in a separate run. Coefficient of Determination (R^2^) of these two sets of data is 0.9922, showing excellent reproducibility. The mean percent difference was 12.7% (Supplementary Figure S1C).

### 2.3 Electronic medical record review

Electronic medical records (EMR) were reviewed in all 212 subjects for diagnostic genetic test results of clinical whole exome sequencing (WES) and/or whole genome sequencing (WGS) and comprehensive mtDNA sequencing in muscle. In addition, routine histopathologic assessment, electron microscopy (EM), mtDNA quantitation, and muscle electron transport chain (ETC) enzyme activity results were reviewed when available. Notably, results of muscle mtDNA content results and ETC enzyme activity were analyzed as counts (categorical data) across “deficiency”, “unremarkable”, or “upregulated” subgroup classifications. In the MLSMD cohort (n = 45), plasma lactate, creatine kinase (CK), growth differentiation factor 15 (GDF15) levels, problem lists, clinical presentations, and the Mitochondrial Myopathy Composite Assessment Tool (MM-COAST) composite scores (Flickinger et al., 2021) at the time of the diagnostic muscle biopsy were manually extracted from the EMR (circled in red, Figure 1).

### 2.4 Statistical analysis

The demographic and clinical characteristics of subjects were summarized by standard descriptive statistics. Fisher’s exact test was used for the comparison of categorical variables among different groups, while Kruskal–Wallis or Student’s t-test was used as appropriate for continuous variables. Cochran-Armitage test for trend was used to assess whether there was an increasing trend in the presence of MLSMD with age. Spearman’s correlation was used to analyze an association of MLSMD heteroplasmy level and age across subjects with different Bernier criteria stratification groups. All analyses were conducted in GraphPad Prism (version 10) or RStudio.

## 3 Results

### 3.1 Study cohort and previous molecular diagnostic testing

The study cohort consisted of 212 subjects, that included 143 (67%) children (<18 years) and 69 (33%) adults. MLSMD were detected in 50 of 212 (24%) diagnostic muscle biopsies that were assessed by the CHOP MitoGenome (Figure 1, Supplementary Table S1). Among these, 47 of 50 (94%) subjects completed clinical WES or WGS. WES/WGS were performed at Clinical Laboratory Improvement Amendments (CLIA) certified diagnostic clinical labs including GeneDx, CHOP, Variantyx, Medical Neurogenetics (MNG), Baylor Genetics, Illumina, or Centogene. All clinical genetic test reports were reviewed, and the results were deemed either diagnostic of a definitive genetic diagnosis or non-diagnostic. One subject (1/50) completed nDNA-based Progressive External Ophthalmoplegia Panel (GeneDx) and mitogenome testing. The remaining two subjects did not have nuclear genomic testing performed due to lack of follow-up in clinic. In the 47 subjects who completed WES and/or WGS, 15 of 47 (32%) had WGS, and 14 of the 15 with WGS (93%) completed WES prior to WGS. This comprehensive clinical genetic diagnostic testing (WES and WGS) is expected to exclude known mtDNA maintenance defect nuclear genes known to be associated with MLSMD in muscle (El-Hattab et al., 2018). Seven of 212 (3%) subjects were found to have a single large-scale mtDNA deletion (SLSMD) and were subsequently excluded from analyses. The remaining 155 of 212 (73%) subjects did not exhibit MLSMD (Figure 1). Within this non-MLSMD cohort, 152 of 155 (98%) subjects completed clinical WES. WGS was also performed in 45 of 152 (30%) subjects.

### 3.2 Subjects with confirmed primary mitochondrial disease (PMD) and other genetic etiologies

Across the study cohort with diagnostic muscle biopsies, a total of 9 of 212 (4%) subjects carried pathogenic or likely pathogenic variants identified in the nuclear or mitochondrial genome consistent with a molecular diagnosis of PMD (Supplementary Table S2). Among 155 subjects without MLSMD, 5 (3.2%) received a PMD genetic diagnosis, while 20 (12.9%) received a non-PMD genetic diagnosis (Figure 1). In the subgroup of 50 subjects with MLSMD, 4 (8%) had a definitive genetic diagnosis of PMD (1 with biallelic pathogenic variants in *C1QBP*, 3 with pathogenic/likely pathogenic mtDNA variants in *MT-TL1, MT-TP, MT-TT*). A fifth subject (P8) with MLSMD had a confirmed diagnosis of myotonic dystrophy type 2 *(DM2*). Indeed, mtDNA deletions have been observed in patients with myotonic dystrophy (Thyagarajan et al., 1993; Sahashi et al., 1992). As our study was focused on subjects with MLSMD without a confirmed molecular diagnosis, all nine subjects with a known PMD genetic diagnosis and the subject with myotonic dystrophy type 2 were excluded from MLSMD cohort analyses (Figure 1 and Supplementary Table S1). The remaining 45/50 subjects with MLSMD did not have a definitive genetic diagnosis identified on clinical diagnostic testing either by WES and/or WGS.

### 3.3 Comprehensive evaluation of MLSMD in muscle tissue

**FIGURE 2:**
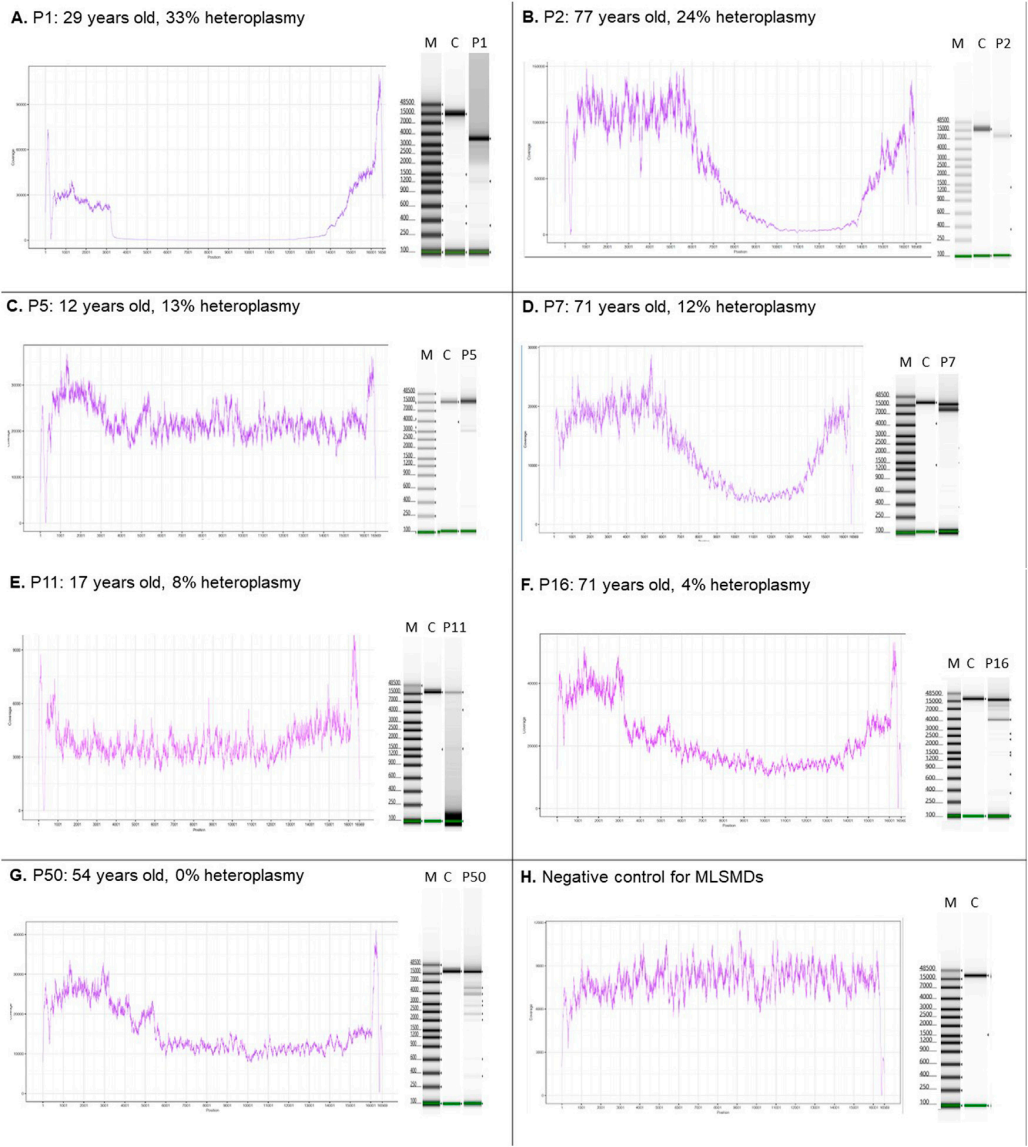
MLSMD detected by long-range PCR and NGS. **(A–D)**. Subjects with MLSMD heteroplasmy ≥10%. **(E–G)**. Three representative subjects with MLSMD heteroplasmy <10%. **(H)** Deletion negative control. Left panels: NGS coverage profile to indicate the presence of large-scale mtDNA deletions. Right panels: long range PCR gel pictures. The presence of multiple amplification bands supports the detection of MLSMD by NGS. M: molecular weight marker; C: controls; P: Subject. X-axis: mtDNA position; Y-axis: NGS coverage depth. The deletion heteroplasmy was evaluated by ddPCR. Heteroplasmy <10% was considered as low level and may not be accurately quantified by ddPCR assay.

The MLSMD detected by NGS were evaluated using laboratory-developed deletion detection plots and LR-PCR gel images (Figure 2). When subjects tested positive for MLSMD, a subsequent ddPCR-based deletion quantification assay was conducted. The heteroplasmy level of MLSMD across the cohort ranged from 0% to 33% as quantified by ddPCR (Supplementary Table S1) noting the constraints of precise ddPCR quantification <10% heteroplasmy levels as described in the Methods Section (2.2). Notably, among the 15 subjects (P36-50) who had MLSMD detected by NGS and supported by LR-PCR gel images, the MLSMD were undetectable by ddPCR, showing 0% heteroplasmy (P50 in Figure 2G as an example). According to serial dilution data (Supplementary Figure S1B), MLSMD heteroplasmy level below 10% cannot be accurately quantified by ddPCR (Figures 2E–G and Supplementary Table S1). These very low-level heteroplasmy deletions fell below the detection limit of ddPCR. Thus, the ddPCR assay may lack sensitivity and precision when quantifying low-level mtDNA deletions. Among the 45 subjects with MLSMD and without genetic diagnosis, 5 (11%) exhibited heteroplasmy levels ≥10%, while the remaining 40 subjects (89%) demonstrated heteroplasmy levels <10% (Supplementary Table S1). Most mtDNA deletion breakpoints were located between positions m.3000_m.15500, consistent with other reported large-scale mtDNA deletion breakpoints (https://www.mitomap.org/MITOMAP). Notably, the common 4977 bp deletion was the most prevalent deletion based on the breakpoints analysis (data not shown).

### 3.4 Age distribution in subjects with and without MLSMD

**FIGURE 3:**
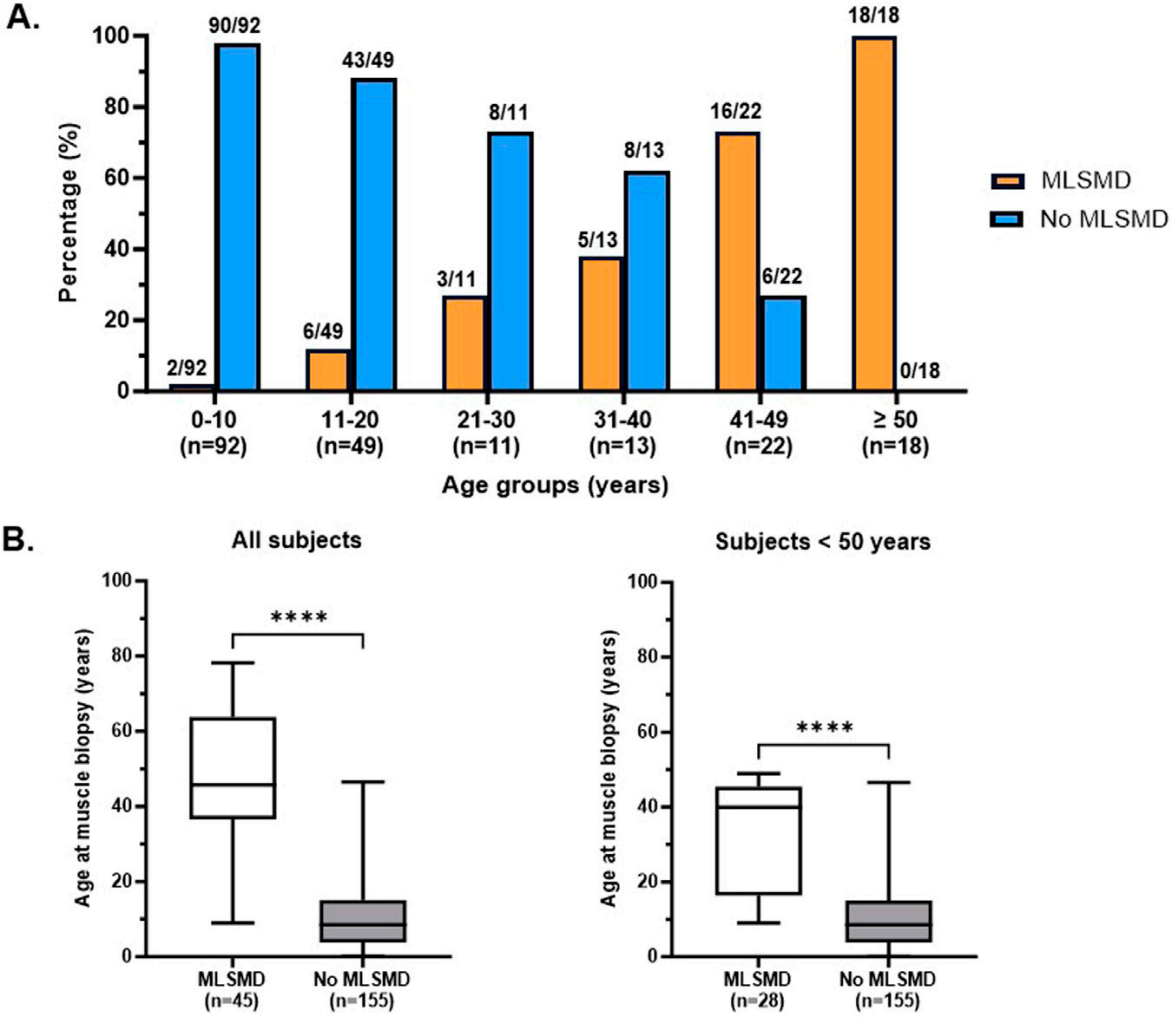
Detection rate of MLSMD across different age groups. **(A)**. Subjects with and without MLSMD were stratified into 10-year age groups. The presence (orange) and lack of (blue) MLSMD subjects were demonstrated in each age group. All subjects in the ≥50 years age group demonstrated MLSMD in their diagnostic muscle biopsies (n = 18). **(B)**. Mean age of the MLSMD cohort (n = 45) was significantly higher as compared to subjects without MLSMD (n = 155), even after comparison of subjects below 50 years of age only (n = 28). ****: p < 0.0001.

Diagnostic muscle biopsies were obtained between 0.1 and 78.2 years of age (19.5 ± 19.4 years, mean ± standard deviation, SD) across the cohort. Among the 212 subjects, 146 (68.9%) were under 20 years of age at the time of biopsy. Notably, all subjects ages ≥50 years at the time of biopsy (n = 18/18) exhibited MLSMD in their skeletal muscle tissue (Figure 3A). A further analysis of subjects stratified into 10-year age groups revealed a significant increasing trend in the presence of MLSMD with age (p-value <0.001, Cochran-Armitage test for trend). Subjects <10 years (2%, n = 2/92) and those subjects between 10 and 20 years (12%, n = 6/49) infrequently exhibited MLSMD (Figure 3A).

**FIGURE 4:**
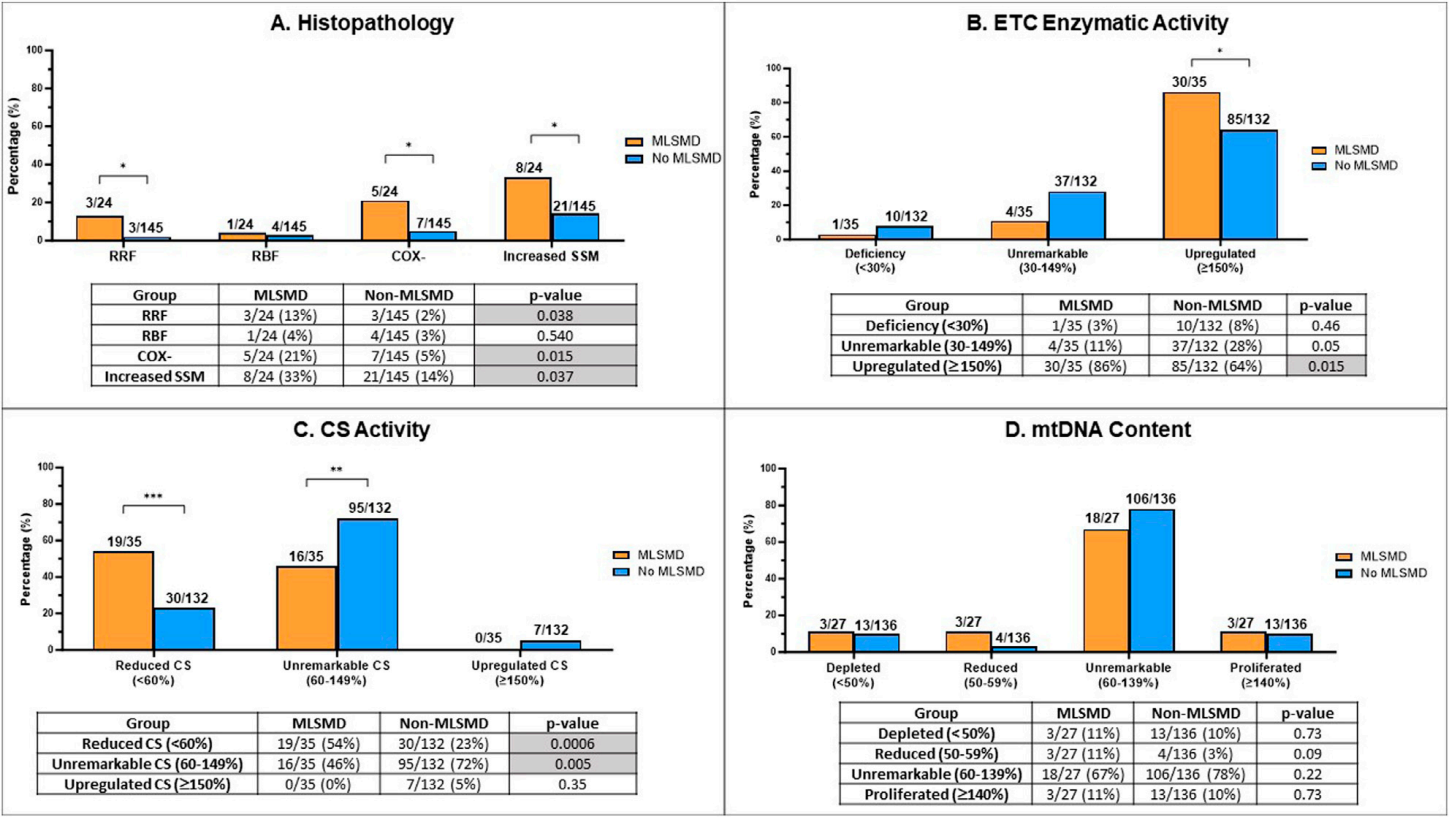
Comparison of muscle biopsy histopathology results, ETC enzyme activity, citrate synthase (CS) activity, and mtDNA content in MLSMD and non-MLSMD groups. Subjects with confirmed PMD were excluded. **(A)** Comparison of the proportion of subjects with ragged red fibers (RRFs), ragged blue fibers (RBFs), cytochrome oxidase negative fibers (COX-), and increased subsarcolemmal mitochondrial (SSM) staining in diagnostic muscle biopsies between MLSMD and the non-MLSMD groups by Fischer’s exact test. Results showed significantly increased subjects with RRFs (p = 0.038), COX- fibers (p = 0.015) and SSM (p = 0.037) in the MLSMD groups as compared to the non-MLSMD groups (<50 years). No statistically significant differences in RBFs were observed between MLSMD and non-MLSMD groups most likely related to the small cohort size. **(B)** Comparison of the proportion of subjects with ETC enzyme activity abnormalities between the MLSMD and non-MLSMD groups. ‘Deficiency’ denotes one or more ETC complex activities meeting the modified Walker diagnostic criteria (<30% of the control value); ‘Unremarkable’ indicates ETC enzyme activities between 30%–149% of the control value; ‘Upregulated’ signifies one or more complex activities ≥150% of the control value. Results showed significantly increased subjects with ‘Upregulated’ ETC enzyme activity in the MLSMD group as compared to the non-MLSMD group (p = 0.015). **(C)** Comparison of the proportion of subjects with abnormal CS activity results between the MLSMD and non-MLSMD groups. ‘Reduced CS’ indicates CS activity <60% of the normal control value; ‘Unremarkable CS’ indicates CS activity between 60%–149% of normal control value; iii) ‘Upregulated CS’ indicates CS activity ≥150% of normal control value. Results showed that subjects with MLSMD had a significantly higher likelihood of having reduced CS activity (p = 0.0006), whereas non-MLSMD subjects had significantly higher likelihood of unremarkable CS activity (p = 0.005). There were no significant differences observed in the ‘Upregulated CS’ category between MLSMD and non-MLSMD groups. **(D)** Comparison of mtDNA content between MLSMD and non-MLSMD groups. ‘Depleted’ indicates mtDNA content <50% of age and tissue matched control values; ‘Reduced’ includes mtDNA content between 50%–59%; ‘Unremarkable’ denotes mtDNA content between 60%–139%; ‘Proliferated’ refers to mtDNA content ≥140% of the age-matched control value. Results showed that the majority of subjects in both the MLSMD and non-MLSMD groups displayed ‘unremarkable’ mtDNA content, which was seen in 67% and 78% of the respective cohorts. No significant differences were observed between mtDNA content results between groups. The bar charts present the rates of abnormal results in the MLSMD and non-MLSMD groups. The corresponding tables show the number of subjects with abnormal results (numerator) and the total number of subjects with available results in each test category (denominator). Fisher’s exact test was used to calculate p-values, determining the significance of the observed differences. *p < 0.05; **p < 0.01; ***p < 0.001.

The mean age of subjects in the MLSMD group at muscle biopsy was 45.1 ± 19.7 years (mean ± SD, n = 45, range 9–78.2 years), which was significantly higher compared to the non-MLSMD subjects at 11.4 ± 10.5 years (n = 155, range 0.1–46.6 years, Figure 3B) (p < 0.0001). To mitigate the confounding effects of age, subjects ≥50 years of age in the MLSMD cohort were subsequently excluded from the inter-cohort (MLSMD vs. non-MLSMD) comparisons (Figure 3, Figure 4). On comparing subjects <50 years in both the MLSMD (n = 28) and non-MLSMD (n = 155) groups, the mean age in the MLSMD group remained significantly higher at 33.3 ± 14.15 years as compared to 11.4 ± 10.5 years in the non-MLSMD group (p < 0.0001) (Figure 3B). Regardless, aging is unlikely to be the sole cause of MLSMD in those subjects <50 years, particularly in the context of their clinical presentations, which suggests the likelihood of an underlying diagnosis.

### 3.5 Inter-cohort comparison between the MLSMD and non-MLSMD cohort

#### 3.5.1 Ragged red fibers (RRFs), ragged blue fibers (RBFs), cytochrome C oxidase negative (COX-) fibers, subsarcolemmal mitochondrial (SSM) accumulation or increased staining on histochemistry

We conducted a comparative analysis of histopathological findings from muscle biopsy diagnostic testing between the MLSMD and non-MLSMD groups, with a specific focus on the presence of RRFs, RBFs, COX- fibers, and SSM accumulation (Figure 4A). Previous studies have reported the association of RRFs and COX-fibers to the aging process (Lu et al., 2019; Larsson, 2010; Kraytsberg et al., 2006). Subjects ages ≥50 years in the MLSMD cohort were excluded from this inter-cohort analysis as there were no subjects ≥50 years in the non-MLSMD cohort. Subjects with genetically confirmed PMD (Supplementary Table S2) in both MLSMD and non-MLSMD cohorts were also excluded from analysis. Additionally, four subjects in the MLSMD cohort (<50 years) and five subjects in the non-MLSMD cohort lacked accessible histopathology reports in their EMR and were excluded. Thus, results from a total of 24 subjects in the MLSMD cohort and 145 subjects in the non-MLSMD cohort were compared. In the MLSMD group, 13% (3/24) of subjects had RRFs, 21% (5/24) had COX- fibers, and 33% (8/24) had SSM accumulation. These frequencies were higher as compared to the non-MLSMD group, where the frequencies were 2% (3/145, p = 0.038) for RRFs, 5% (7/145, p = 0.015) for COX- fibers, and 14% (21/145, p = 0.037) for SSM accumulation. Available reports of RBFs were limited. RBFs were similarly observed in the MLSMD and non-MLSMD groups, at 4% (1/24) and 3% (4/145), respectively (p = 0.54, Fisher’s exact test; Figure 4A).

#### 3.5.2 Muscle ETC enzymatic activities assay and mtDNA content

Subjects of all ages were included in this analysis, but subjects with confirmed PMD were excluded (Supplementary Table S2). Results of the muscle ETC enzymatic activities assay were categorized into three groups (Figure 4B) consisting of i) “deficiency”, defined as a decrease in one or more ETC enzyme complex activities that met the modified Walker diagnostic criteria (<30% of the control value) (Bernier et al., 2002; Walker et al., 1996); ii) “unremarkable”, defined as citrate synthase (CS)-corrected ETC enzyme activities between 30%–149% of the control value; and iii) “upregulated”, defined as an increase ≥150% of one or more corrected ETC enzyme activities. CS activity, a widely used quantitative enzyme marker, is utilized to assess the presence of intact mitochondria (Vigelsø et al., 2014). We grouped CS results into three categories (Figure 4C) consisting of i) reduced CS activity (<60% of control values); ii) unremarkable CS (60%–149% of control); iii) upregulated CS (≥150% of control). In our definitions, the thresholds for “reduced” and “upregulated” CS were based on ± 2 SD as provided by the clinical laboratories.

Results revealed that subjects with MLSMD with available results (n = 30/35, or 86%) were significantly more likely to have “upregulated (≥150%)” muscle ETC enzymatic activity results compared to the non-MLSMD subjects (n = 85/132, 64%), p = 0.015 by Fischer’s exact test (Figure 4B). However, there were no significant differences observed in the “deficiency” (3% in MLSMD vs. 8% in non-MLSMD, p = 0.46) or “unremarkable” categories (11% in MLSMD vs. 28% in non-MLSMD, p = 0.05). These results indicate that muscle ETC enzyme activity deficiencies meeting modified Walker criteria (Walker et al., 1996; Bernier et al., 2002) were not more frequently observed in the MLSMD group as compared to the non-MLSMD groups. However, upregulated ETC enzyme activities were more frequently observed.

Subjects with MLSMD were more likely to have “reduced” CS as compared to non-MLSMD subjects, 54% vs. 23%, p = 0.0006 by Fischer’s exact test; while non-MLSMD subjects were more likely to have “unremarkable” CS as compared to MLSMD subjects, 72% vs. 46%, p = 0.005. There were no significant differences in the “upregulated” CS’ category between MLSMD and non-MLSMD groups. Notably, none of the MLSMD subjects met criteria for “upregulated” CS (Figure 4C).

Since mtDNA content was measured using different quantitative PCR (qPCR) methods by two diagnostic laboratories (Baylor Genetics and CHOP), the raw mtDNA copy numbers could not be directly compared. Results of mtDNA content were grouped into four categories consisting of i) “depleted” as defined by mtDNA content <50% of age- and tissue-matched controls; ii) “reduced”, defined as 50%–59% of control values; iii) “unremarkable” with mtDNA content between 60%–139%; and iv) “proliferated” where mtDNA content ≥140% of age-matched control values. These thresholds were based on the diagnostic laboratory classifications for mtDNA depletion and utilized approximately +2 SD to define proliferation. Most subjects in both the MLSMD and non-MLSMD groups had “unremarkable” mtDNA content, which was seen in 67% and 78% of the respective cohorts (p = 0.22, Fisher’s exact test). No significant differences were observed in any mtDNA content categories in the MLSMD vs. non-MLSMD group (Figure 4D).

### 3.6 Intra-cohort MLSMD subject analysis

#### 3.6.1 Subjects with MLSMD (n = 45) stratified by likelihood of PMD diagnosis

**FIGURE 5:**
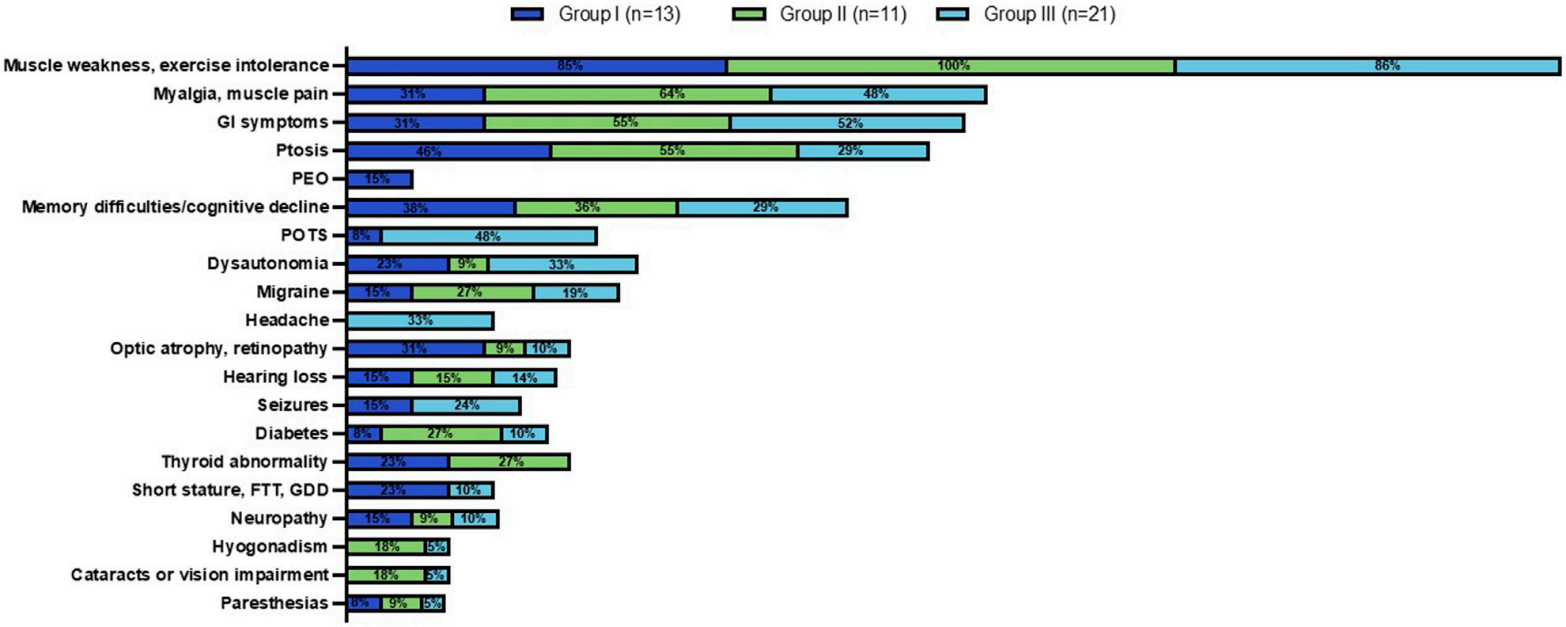
Clinical phenotype across MLSMD Groups I-III. Group I comprised of subjects with a high suspicion of PMD. Group II included subjects age ≥50 years with a lower index of suspicion, classified as ‘Less likely PMD’. The remaining subjects in Group III were <50 years of age, also considered as ‘Less likely PMD’. The percent (%) in each colored box represents the number of subjects with the stated phenotype/total subjects in Groups I (dark blue), II (green) or III (light blue, see key). Muscle weakness and exercise intolerance was consistently the most prevalent symptom across all three groups (85%–100%). Other common features observed across all three groups included myalgias/muscle pain, gastrointestinal (GI) symptoms, ptosis, and memory difficulties/cognitive decline. PEO: Progressive external ophthalmoplegia; POTS: Postural orthostatic tachycardia syndrome; FTT: Failure to thrive; GDD: Global developmental delay.

The clinical features across all subjects with MLSMD were broadly heterogenous. We classified the 45 subjects with MLSMD who lacked a molecular diagnosis into three distinct subgroups (Groups I-III) based on the likelihood of a PMD diagnosis, using the major and minor symptoms of Bernier criteria (Bernier et al., 2002) (Figure 5). Group I consisted of subjects with a high suspicion of PMD, classified as “Probable/Possible” by Bernier criteria, and subsequently referred to as “suspected PMD” (n = 13, mean age 43.7 years) in this manuscript. The remaining subjects in Group II (50 years and older) and III (<50 years of age) met Bernier criteria for the “Unlikely PMD” category, who we referred to as “Less likely PMD” on close review and interpretation of their clinical phenotypes. Our overall clinical impression of Group II subjects is that they have a lower probability of having PMD and may harbor a genetic neuromuscular disorder or other secondary cause of mitochondrial dysfunction (n = 11). Subjects in Group III were <50 years of age and also were likely to have other genetic or non-genetic etiologies of mitochondrial dysfunction (n = 21).

#### 3.6.2 Clinical features in subjects with MLSMD without genetic etiology (n = 45)

We reviewed the documented clinical features at presentation in the EMR of all MLSMD subjects (Figure 5). Muscle weakness and exercise intolerance were consistently the most common symptoms across all three subgroups (Groups I through III), occurring in 85%–100% of subjects. Other frequent symptoms included myalgias/muscle pain, gastrointestinal issues, ptosis, and memory difficulties/cognitive decline, which were observed in all groups. Despite some overlap in clinical features, distinct symptoms were more common in Group I with “suspected” PMD. Specifically, progressive external ophthalmoplegia (PEO), a feature more specific to PMD, was found exclusively in Group I. Optic atrophy, retinopathy, and short stature were also more prevalent in Group I compared to Groups II and III (“less likely PMD”). In contrast, postural orthostatic tachycardia syndrome (POTS) and headaches were primarily observed in Group III, indicating more non-specific clinical presentations in this subgroup. Ptosis was most frequently observed in Group II, where a genetic neuromuscular diagnosis was suspected in most subjects (Figure 5).

Subjects were further evaluated using the Mitochondrial Myopathy Composite Assessment Tool (MM-COAST) (Flickinger et al., 2021), a validated and objective measure of mitochondrial myopathy. MM-COAST is a comprehensive assessment developed to quantify key myopathy deficits, including muscle weakness, muscle fatigue, exercise intolerance, imbalance, and poor dexterity—all of which are common in mitochondrial myopathy. Higher MM-COAST scores indicate greater severity of symptoms. Among the subjects in this study who completed the MM-COAST assessment, the mean MM-COAST composite score was 0.79 ± 0.25 for Group I (mean ± SEM, range −0.53 to 2.17, n = 11). For Group II, the mean composite score was −0.03 ± 0.17 (range −0.38 to 0.42, n = 4), and for Group III, the mean composite score was 0.33 ± 0.3 (range −0.67 to 1.75, n = 8). While there were no statistically significant differences among the three subgroups (Kruskal–Wallis test, p = 0.18), previously published mean MM-COAST scores were 1.3 ± 0.1 (range 1.04–1.50, n = 53) for a genetically-confirmed PMD cohort and 0.5 ± 0.2 (range 0.14–0.84, n = 29) for a cohort without PMD genetic etiology (Flickinger et al., 2021). The higher MM-COAST scores in Group I support the likelihood that some subjects in Group I may have a PMD diagnosis that thus far has not been identified despite comprehensive diagnostic testing (Table 1).

**TABLE 1:** MLSMD cohort MM-COAST scores, biochemical markers, and muscle biopsy results.

MM-COAST Data				
	Group I (n = 13, 11 with data)	Group II (n = 11, 4 with data)	Group III (n = 21, 8 with data)	Kruskal Wallis
MM-COAST Composite Score mean ± SEM (range)	0.79 ± 0.25	−0.03 ± 0.17	0.33 ± 0.30	p = 0.18

MLSMD Cohort Biochemical Markers				
	Group I	Group II	Group III	Kruskal Wallis
Mean plasma lactate ± SEM (range)	2.29 ± 0.89 (0.61–12.3)	1.23 ± 0.21 (0.2–2.39)	1.1 ± 0.1 (0.6–2.08)	p = 0.46
Mean CK ± SEM (range)	588.3 ± 232.1 (45–2,351)	104.23 ± 15.2 (50–233)	204.2 ± 76.1 (20–1,554)	p = 0.18
Mean GDF15 ± SEM (range)	1,839.4 ± 437.8 (373–4,558)	1,051 ± 110.5 (507–1831)	682.3 ± 89.4 (301–1773)	p = 0.04
Subjects with GDF15 > 1200 pg/mL	5/11 (45%)	1/9 (11%)	1/12 (8%)	p = 0.10

MLSMD Cohort (n=45, 40/45 with available histology report)^a^				
	Group I (n = 13, 12 with reports)	Group II (n = 11, 10 with reports)	Group III (n = 21, 18 with reports)	Two-sided Fisher’s Exact
Ragged Red Fibers	5/12 (42%)	0/10 (0%)	1/18 (6%)	p = 0.01
Ragged Blue Fibers	2/12 (17%)	0/9 (0%)	0/18 (0%)	p = 0.14
COX - Fibers	6/12 (50%)	8/10 (80%)	3/18 (17%)	p = 0.004
Type I Predominance	3/11 (27%)	0/10 (0%)	1/18 (6%)	p = 0.14
Type II Predominance	3/11 (27%)	5/10 (50%)	7/18 (39%)	p = 0.58
Fiber Type Grouping	2/11 (18%)	2/9 (22%)	1/18 (6%)	p = 0.48
Variation in Fiber Size	9/12 (75%)	6/10 (60%)	9/18 (50%)	p = 0.46
Internally placed nuclei	6/12 (50%)	1/10 (10%)	3/18 (17%)	p = 0.08
Nuclear Bags	2/12 (17%)	2/10 (20%)	0/18 (0%)	p = 0.10
Fiber Degeneration and Regeneration	2/12 (17%)	1/10 (10%)	0/18 (0%)	p = 0.16
Perivascular Inflammation	0/12 (0%)	0/10 (0%)	0/18 (0%)	p = 1

MLSMD Cohort ETC Enzyme Activity Analyses				
	Group I (n = 13, 9 with reports)	Group II (n = 11, 9 with reports)	Group III (n = 21, 17 with reports)	Two-sided Fisher’s Exact
Deficiency (<30%)	1/9 (11%)	0/9 (0%)	0/17 (0%)	p = 0.51
Unremarkable (30%–149%)	0/9 (0%)	1/9 (11%)	3/17 (18)	p = 0.79
Upregulated (≥150%)	8/9 (89%)	8/9 (89%)	14/17 (82%)	p = 1

MLSMD Cohort Citrate Synthase Activity Analyses				
	Group I (n = 13, 9 with reports)	Group II (n = 11, 9 with reports)	Group III (n = 21, 17 with reports)	Two-sided Fisher’s Exact
Reduced CS (<60%)	3/9 (33%)	7/9 (78%)	9/17 (53%)	p = 0.19
Unremarkable CS (60%–149%)	6/9 (67%)	2/9 (22%)	8/17 (47%)	p = 0.19
Upregulated CS (≥150%)	0/9 (0%)	0/9 (0%)	0/17 (0%)	p = 1

MLSMD mtDNA Content Analyses				
	Group I (n = 13, 7 with reports)	Group II (n = 11, 5 with reports)	Group III (n = 21, 15 with reports)	Two-sided Fisher’s Exact
Depleted (<50%)	2/7 (29%)	0/5 (0%)	1/15 (7%)	p = 0.23
Reduced (50%–59%)	0/7 (0%)	0/5 (0%)	3/15 (20%)	p = 0.39
Unremarkable (60%–139%)	4/7 (57%)	5/5 (100%)	9/15 (60%)	p = 0.26
Proliferated (≥140%)	1/7 (14%)	0/5 (0%)	2/15 (13%)	p = 1

^a^ Histopathologic evaluation of muscle biopsies had been performed at multiple institutions for clinical purposes. Precise quantification of pathologies (such as RRF, COX- fibers) was not included in the reviewed pathology reports. Therefore, we documented these histologic features as present/absent.

CK: creatine kinase; GDF15: growth differentiation factor 15; COX- fibers: cytochrome oxidase negative fibers; ETC: electron transport chain; CS: citrate synthase.

#### 3.6.3 Biochemical laboratory measurements and muscle histopathology in MLSMD cohort

**FIGURE 6:**
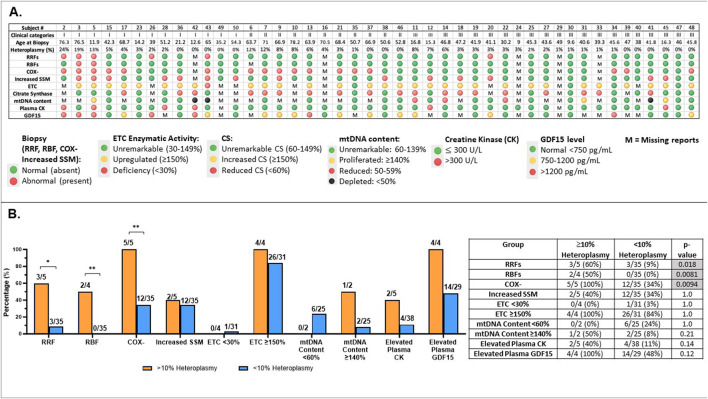
Comprehensive analysis of subjects with MLSMD (n = 45) and correlation with heteroplasmy levels **(A)**. Results overview for 45 subjects with MLSMD lacking a genetic diagnosis. Results display individual subject demographics, histopathologic, and biochemical characteristics across the MLSMD cohort. Subject # corresponds to the subject list in Supplementary Table S1. Five subjects with known PMD genetic diagnosis (or DM2) were excluded from these analyses (Supplementary Table S1). MLSMD heteroplasmy levels were detected and quantified by combined NGS, LR-PCR, and ddPCR analysis. Please note, heteroplasmy levels <10% are below ddPCR detection limit and therefore may not be accurately quantified by ddPCR, but can be detected by NGS and LR-PCR. M: Missing report or no result available. **(B)**. Abnormal results identified in MLSMD cohort by mtDNA heteroplasmy levels (≥10% and <10%). **(B)** illustrates the rates of abnormal results in histopathology (RRFs: ragged red fibers; RBFs: ragged blue fibers; COX-: cytochrome oxidase negative fibers; Increased SSM: Increased subsarcolemmal mitochondrial staining/accumulation), muscle electron transport chain (ETC) assay, mtDNA content, Creatine kinase (CK), and growth differentiation factor 15 (GDF15) in each test category, distinguishing between MLSMD heteroplasmy levels of 10% and above and those below 10%. The number of subjects with abnormal results, the total number of subjects with available results in each diagnostic test category, and the ’abnormal percentages’ are listed in the table. Histological abnormalities including RRFs, RBFs, and COX– fibers occurred significantly more frequently in the ≥10% heteroplasmy groups when compared to the <10% heteroplasmy groups (p = 0.018, p = 0.0081, p = 0.0094, respectively by Fisher’s exact test). *p-value <0.05, **p-value <0.01.

Results of muscle histopathology, muscle ETC enzyme activity, muscle mtDNA content measurement, as well as plasma lactate, CK, and GDF15 across the MLSMD group were reviewed (Figure 6A) and compared between subjects with higher MLSMD heteroplasmy (≥10%, n = 5) as compared to low-level heteroplasmy (<10%, n = 40) (Figure 6B).

The distribution of subject heteroplasmy levels (Figure 6A, Supplementary Table S1) across the MLSMD group included 5/45 (11%) subjects with heteroplasmy levels ≥10%, 4/5 subjects were ≥50 years of age. There were 3/5 (60%) subjects with heteroplasmy levels ≥10% who were in Group 1 “suspected” PMD, 2/3 of these subjects were ≥50 years old; and the remaining 2/5 (40%) subjects were in Group II. All subjects in Group III (n = 21) had <10% heteroplasmy levels (Figure 6A). Histological abnormalities including RRFs, RBFs, and COX– fibers occurred significantly more frequently in the ≥10% heteroplasmy group (n=5) as compared to the <10% heteroplasmy groups (n=40), p = 0.018, p=0.0081, p=0.0094, respectively, by Fisher’s exact test. However, the sample size of the ≥10% heteroplasmy group (n =5) is limited and should be interpreted with caution (Figure 6B).

Biochemical diagnostic test results were compared across Groups I, II, and III (Table 1). Mean plasma CK levels trended higher in Group I (588 ± 232.1 U/L, mean ± SEM, n = 12) as compared to Groups II (104 ± 15.2 U/L, n = 11) and III (204 ± 76.1 U/L, n = 20), (Kruskal-Wallis test, p = 0.18). There was also no significant difference in baseline serum lactate levels across Groups I–III, although the mean plasma lactate in Group I was 2.29 ± 0.89 (0.61–12.3 mM, n = 13) as compared to 1.23 ± 0.21 (0.2–2.39 mM, n = 11) in Group II and 1.1 ± 0.1 (0.6–2.08 mM, n = 21) in Group III, p = 0.46, and likely did not reach significance due to the broad range (0.61–12.3 mM) in Group I.

In contrast, mean plasma GDF15 values were significantly higher in Group I (1,839 ± 437.8 pg/mL, n = 11) and Group II (1,051 ± 110.5 pg/mL, n = 9), both exceeding the normal reference range of <750 pg/mL, compared to Group III (682 ± 89.4 pg/mL, n = 12), p = 0.04. Published studies have indicated that GDF15 values greater than 1,200 pg/mL are useful for distinguishing mitochondrial myopathy from other types of metabolic myopathies and muscular dystrophies (Poulsen et al., 2020).

Salient histopathological results included a significantly increased observation of RRF (p = 0.01) in Group I (n = 5/12, 42%) as compared to Groups II (n = 0/10, 0%) and III (n = 1/18, 6%), and a significantly increased frequency of COX- fibers (p = 0.004) in Group I (6/12, 50%) and II (8/10, 80%), compared to Group III (3/18, 17%) by Fisher’s exact test. COX- fibers can be seen as part of the aging process and the significance of this histopathologic finding should be interpreted in the context of the individual’s age (Chariot et al., 1996; Rifai et al., 1995). The remaining histopathological findings listed in Table 1 did not reach significance. Notably, subjects with internally placed nuclei (Koenig, 2008; Pesce et al., 2001) trended higher in Group I (6/12, 50%) when compared to Groups II (1/10, 10%) and III (3/18, 17%), p = 0.08. Similarly, there was a trend for more subjects with myopathic features such as degeneration/regeneration, type I fiber predominance, and neurogenic changes like fiber-type grouping and nuclear bags in Group I. However, these histological findings are non-specific and can be observed in various myopathies.

Additionally, in the majority of subjects across Groups I–III, muscle ETC enzyme activity measurements and mtDNA content results were not diagnostically abnormal (Table 1). Only one (n = 1/35 subjects with reports available) had an ETC enzyme activity deficiency meeting Walker criteria (<30%), and three (n = 3/27 subjects with reports available) met criteria for mtDNA depletion (<50%). There were no significant differences in ETC enzyme activity and mtDNA content results across Groups I–III (Table 1). Interestingly, upregulated CS activity (≥150%) was not observed in any subjects in the MLSMD cohort. These results highlight the importance of integrating biochemical, histological, and clinical assessments in the clinical diagnostic assessment for PMD. A future multi-center study with a larger MLSMD cohort would provide more reliable evidence of an association between MLSMD and histopathologic and biochemical abnormalities. However, we must emphasize that histopathologic and biochemical abnormalities are not confirmatory of PMD. Ultimately, genetic testing is required to confirm a definitive diagnosis of PMD (Ng et al., 2021; Mccormick et al., 2018).

In summary, the clinical features, MM-COAST scores, histopathological findings (such as RRF and COX- fibers), and GDF15 levels in Group I supported a higher likelihood of a PMD diagnosis as compared to Groups II and III. In contrast, subjects in Groups II and III revealed a relatively high frequency of non-diagnostic abnormalities in their histopathologic, muscle ETC, and biochemical laboratory tests. This suggests that other non-PMD genetic etiologies or non-genetic factors may be associated with MLSMD in these subjects.

## 4 Case vignettes to highlight potential for misinterpretation of MLSMD for single, large-scale mtDNA deletion

Two subjects in the MLSMD cohort, P11 and P50 (Figure 2E, G), were previously reported as harboring a single large-scale mitochondrial DNA deletion (SLSMD) by external diagnostic laboratories. Repeat mtDNA sequencing in muscle was performed at CHOP to better assess heteroplasmy levels. The CHOP Mitogenome analyses in both subjects confirmed MLSMD rather than a SLSMD. Upon retrospective review, although both subjects exhibited ptosis and musculoskeletal symptoms that could be associated with SLSMD syndrome (SLSMDS), they lacked other key features of SLSMDS. Specifically, neither subject had progressive external ophthalmoplegia (PEO), and neither showed hallmark findings of mitochondrial myopathy on their muscle biopsy histopathology.

Given the universal presence of MLSMD in our research cohort age ≥50 years and the aforementioned misdiagnosis of MLSMD as SLSMD, we reviewed our clinical cohort of SLSMDS for subjects ≥50 years to assess for other potentially misdiagnosed cases. Only one subject ≥50 years of age was identified in our clinical cohort with a diagnosis of SLSMDS. This subject had CPEO, COX-fibers and evidence for denervation with secondary myopathic changes on muscle histology. Combined mitogenome and mitochondrial myopathy nuclear gene panel (GeneDx) performed in blood was normal. The plasma GDF15 level was elevated at 2653 pg/mL (normal reference range <750 pg/mL). Mitogenome analysis in this subject’s muscle obtained at age 55.5 years was performed at an external diagnostic laboratory, and thus excluded from primary analysis (Figure 1). The muscle biopsy for this subject revealed the common 4977 bp single large-scale mtDNA deletion through both Southern blot and NGS, confirming a SLSMDS diagnosis. Additionally, MLSMD were also identified in this individual, only by NGS but not by Southern blot. While the clinical report for this subject’s mitogenome analysis in muscle does not provide specific heteroplasmy levels of the MLSMD, their detection by NGS but not by Southern blot suggests they are present at low level. This case highlights an example of SLSMD coexisting with likely age-related MLSMD.

## 5 Discussion

Results of our study describe the clinical, biochemical, and histopathological characteristics of 45 subjects with MLSMD without definitive genetic etiologies. It is crucial to emphasize that this cohort of subjects with MLSMD does not represent a single clinical entity or phenotype. MLSMD in muscle tissue may be associated with a broad range of heterogeneous etiologies including PMD, other genetic disorders, aging, and non-genetic factors. In regards to PMD, MLSMD are regarded as hallmark indicators of mtDNA replication and maintenance defects caused by pathogenic variants in nuclear genes (Ahmed et al., 2015; Bychkov et al., 2021; Carrozzo et al., 1998; Lujan et al., 2020). However, in our study cohort of 50 subjects with MLSMD in muscle tissue, only one individual exhibited pathogenic variants in *C1QBP* known to be associated with MLSMD (Wilcox et al., 2022). No causal PMD nuclear gene defects associated with MLSMD were identified on WES and/or WGS in the remaining 49 subjects with MLSMD, in whom 3/49 subjects harbored mtDNA genetic etiologies of PMD and 1 caried a myotonic dystrophy type 2 diagnosis without co-existing PMD nuclear or mtDNA genetic etiology.

To explore the relationship between the presence of MLSMD and the likelihood of PMD, we retroactively applied the major/minor symptoms of Bernier criteria to each subject. This delineation identified 13 of 45 subjects in Group I (28.9%) having a high likelihood of PMD, categorizing them as “suspected PMD” in alignment with our clinical impressions at their clinic visits. Indeed, Group I showed a significantly higher frequency of RRF (p = 0.01), COX- fibers (p = 0.004), and elevated plasma GDF15 levels (p = 0.04) compared to the “less likely PMD” subjects categorized in Groups II and III, along with higher MM-COAST scores (Flickinger et al., 2021) that were distinct from the MM-COAST composite scores observed in Groups II and III subjects. The integration of clinical, histopathological, and biochemical evaluations is invaluable in the diagnostic assessment of PMD. These results highlight the importance of ongoing clinical and research evaluations in Group I subjects identified with MLSMD in muscle tissue who express a constellation of clinical features that raise high suspicion for PMD. However, while the integration of these clinical, histopathological, and biochemical results may provide critical supportive evidence for a PMD diagnosis, they do not confirm a definitive diagnosis of PMD, nor do they provide prognostic information. Indeed, a pathogenic etiology at the gene and variant level is now required to definitively confirm a diagnosis of PMD (Mccormick et al., 2018). Ongoing follow-up in a dedicated Mitochondrial Medicine clinical center that implements a systematic approach to clinical and research-level diagnostic expertise remains essential to continue to identify novel genetic etiologies causal of PMD.

There are limited mechanistic studies characterizing the generation of mtDNA deletions (Fontana and Gahlon, 2020). The shift in single large-scale mtDNA heteroplasmy over time and across tissue types has been previously described (Grady et al., 2014; Stewart and Chinnery, 2015). Indeed, characterizing genotype phenotype correlations for a single large scale mtDNA deletion as occurs in SLSMDS is complex (Grady et al., 2014). Therefore, predicting the clinical phenotype and impact on the mitochondrial respiratory chain function based on an individual’s MLSMD deletion breakpoints would be challenging due to several potentially correlated and confounding variables that include i) the lack of causative genetic etiologies across our MLSMD cohort, ii) potential heteroplasmy shift over time, and iii) the overlap in multiple mtDNA deletions potentially leading to the deleted region of one mtDNA molecule being complemented by the non-deleted region of another mtDNA molecule. Furthermore, there is also the contributory effect of the loss of tRNA genes on mitochondrial protein synthesis, which may vary across multiple deletions. Thus, there are numerous variables to account for when considering the impact of specific mtDNA deletions breakpoints on the clinical and biochemical phenotype.

Human aging has been linked to the accumulation of somatic mtDNA mutations and a decline in mitochondrial respiratory chain function (Larsson, 2010; Zhang et al., 1992; Bender et al., 2006). As individuals age, somatic MLSMD accumulate in post-mitotic tissues, such as muscle (Larsson, 2010; Herbst et al., 2021b; Herbst et al., 2021a). Our study indeed revealed that all subjects aged 50 years and older at time of biopsy (n = 18) exhibited MLSMD in their muscle tissues. Further, there was an increasing trend in the presence of MLSMD with age (Figure 3). It is important to note that, while we observed an association between MLSMD and aging, the subjects in our study cohort were not asymptomatic, healthy individuals. Specifically, these subjects expressed musculoskeletal and/or other organ system involvement. Given their extensive symptoms, it is unlikely that the observed muscle MLSMD detected in these older subjects can be fully attributed to the normal aging process. In contrast, in our younger study population, MLSMD were observed in two subjects in the range of 0–10 years of age and six subjects in the range of 11–20 years (Figure 3A, Supplementary Table S1). Of these eight subjects, three were in Group I with a high suspicion of PMD. Aging would not be a causative factor for MLSMD in these younger subjects, thus positioning them as candidates for genomic research studies to identify novel genetic etiologies associated with muscle MLSMD.

The ultra-high coverage depth in LR-PCR based NGS sequencing for mtDNA analysis can detect MLSMD below 10% heteroplasmy. However, it does not allow for precise quantification of the heteroplasmy level due to the preferential amplification by LR-PCR of smaller mtDNA molecules, including mtDNA with large-scale deletions. This results in an over-estimation of the true heteroplasmy level, which can be advantageous in some regards as it increases the sensitivity for detecting low-level MLSMD. Therefore, a separate assay, such as ddPCR, is required for accurate MLSMD heteroplasmy quantification. While not as sensitive as NGS, ddPCR remains a sensitive assay for mtDNA deletion heteroplasmy quantification at heteroplasmy levels ≥10% (Wang et al., 2022) and is widely used in research and clinical testing. Other approaches for deletion heteroplasmy quantification include restriction fragment length polymorphism (RFLP), array comparative genomic hybridization (aCGH), and multiplex ligation-dependent probe amplification (MLPA). However, each of these methods has limitations and is generally even less sensitive than ddPCR at detecting low-level (<10% heteroplasmy) MLSMD (Wang et al., 2022).

Nonetheless, the ddPCR assay has its limitations. Our data showed that LR-PCR and deep NGS coverage identified very low-level (<10% heteroplasmy) MLSMD which could not be accurately quantified, or even detected, by ddPCR (Figures 2E–G and Supplementary Table S1, Supplementary Figure S1). Thus, the ddPCR assay lacks sufficient sensitivity to quantify these very low-level deletions. In our clinical diagnostic reports of the CHOP MitoGenome, we report low-level mtDNA deletions detected by high-sensitivity NGS as heteroplasmy values < 10% without providing a precise heteroplasmy level. It would be critical to highlight that the clinical significance of low level MLSMD <10% in informing the diagnostic evaluation and prognosis in a patient’s evaluation for PMD is uncertain. It is noteworthy that 10/13 (76.9%) subjects in MLSMD Group 1 “suspected PMD” had <10% heteroplasmy levels. Further studies of larger MLSMD cohorts are needed.

The ability to clearly distinguish between MLSMD and SLSMD (Wang et al., 2022), which are two distinct genetic etiologies, is also of critical importance. Accurate ascertainment of MLSMD vs. single large-scale mtDNA deletion syndrome (SLSMDS) would be critical for patient management, genetic counseling, and appropriate enrollment for clinical intervention trials. Our clinical case was not included in the primary analysis due to a bona-fide SLSMDS diagnosis and co-existing, likely age-related, MLSMD. This case highlights the complexity of diagnosing and interpreting MLSMD. Indeed, as was highlighted in two of our MLSMD cohort subjects (P11 and P50), MLSMD have been mistaken for a SLSMD. However, the two can also clearly co-exist in older individuals (unpublished data in our clinic cases) and in the literature (Ascaso et al., 2010).

Results of our study highlight important considerations in the clinical interpretation of observed MLSMD in muscle. First, MLSMD were universally observed in individuals ≥50 years, in keeping with the known contribution of age to MLSMD in muscle tissue. Second, the MLSMD in Group II subjects ≥50 years may be associated with novel neuromuscular genetic etiologies causative of their neuromuscular symptoms that have not thus far been identified. Third, MLSMD do not represent a single diagnostic entity as the MLSMD cohort in this study were of varying clinical, biochemical, and histopathologic phenotypes. Fourth, subjects in Group I showed compelling clinical and biochemical phenotypes of PMD and are optimal candidates for research genome analysis and novel gene discoveries, particularly those <50 years. As the genetic etiologies for the MLSMD in these subjects is lacking, our understanding of the potential molecular mechanisms leading to MLSMD is limited. These Group I MLSMD subjects should continue to be followed in clinic. Fifth, future studies with larger cohort sizes of the ≥10% and <10% heteroplasmy MLSMD groups would be necessary to further evaluate the significantly higher rate of muscle histopathological abnormalities in the ≥10% heteroplasmy group that was observed in this study. Lastly, MLSMD is an entirely separate diagnostic entity from SLSMDS. Misinterpretation of MLSMD as SLSMD can occur and is potentially detrimental to patient/family genetic counseling and clinical management.

This study had several limitations. i) A healthy control comparator group was lacking, as individuals without muscle symptoms do not undergo muscle biopsy. ii) Our sample size was limited to subjects who completed muscle mitogenome sequencing at our institution because external muscle mtDNA sequencing raw data were not accessible for verification of results, and other diagnostic laboratories utilized different methods that may not be as sensitive in detecting MLSMD. We recognize that results of our study may not fully represent our clinic cohort who had diagnostic muscle biopsies in its entirety. Future multi-center studies with a larger cohort of subjects with MLSMD are needed. iii) Our analysis did not involve a close review of medications or environmental exposures that could be linked to MLSMD due to limitations of EMR documentation or insufficient records. iv) Another critical limitation is that the overall mean ages as in the MLSMD cohort of 45.1 years (prior to exclusion of subjects ≥50 years) and 33.5 years (after exclusion of subjects ≥50 years), were higher as compared to the non MLSMD cohort with a mean age of 11.3 years (Figure 3B). Subjects ≥50 years were excluded from some analyses to account for age, as age-related histopathological findings have been previously reported (Herbst et al., 2021a; Crane et al., 2010). Future studies are planned to address these limitations.

In summary, this is the first study to characterize a cohort of individuals with MLSMD in muscle tissue of undefined etiologies. Given the myriad of PMD and non-PMD causes of MLSMD reported in the literature, the etiologies across our MLSMD cohort remain elusive in light of their negative comprehensive genetic diagnostic testing. However, in our cohort of 45 subjects with genetically undiagnosed MLSMD, a subset in Group I was classified as “suspected” PMD based on their clinical phenotype and indeed demonstrated a higher likelihood of abnormalities across their biochemical (GFD15) and diagnostic muscle biopsy testing including histopathology (RRFs, COX- fibers), and MM-COAST scores (Flickinger et al., 2021) that were distinct from Groups II and III composite scores. This Group I cohort of subjects with MLSMD in muscle tissue represents a promising cohort for novel gene discoveries and should continue to be diagnostically evaluated.

## Data Availability

The original contributions presented in the study are included in the article/Supplementary Material, further inquiries can be directed to the corresponding authors.
