# Supplementary figures and images for: Interpreting the clinical significance of multiple large-scale mitochondrial DNA deletions (MLSMD) in skeletal muscle tissue in the diagnostic evaluation of primary mitochondrial disease
Source: Front Pharmacol. 2025 Apr 9;16:1507493. doi: 10.3389/fphar.2025.1507493 (PMC12015102; doi:10.3389/fphar.2025.1507493)

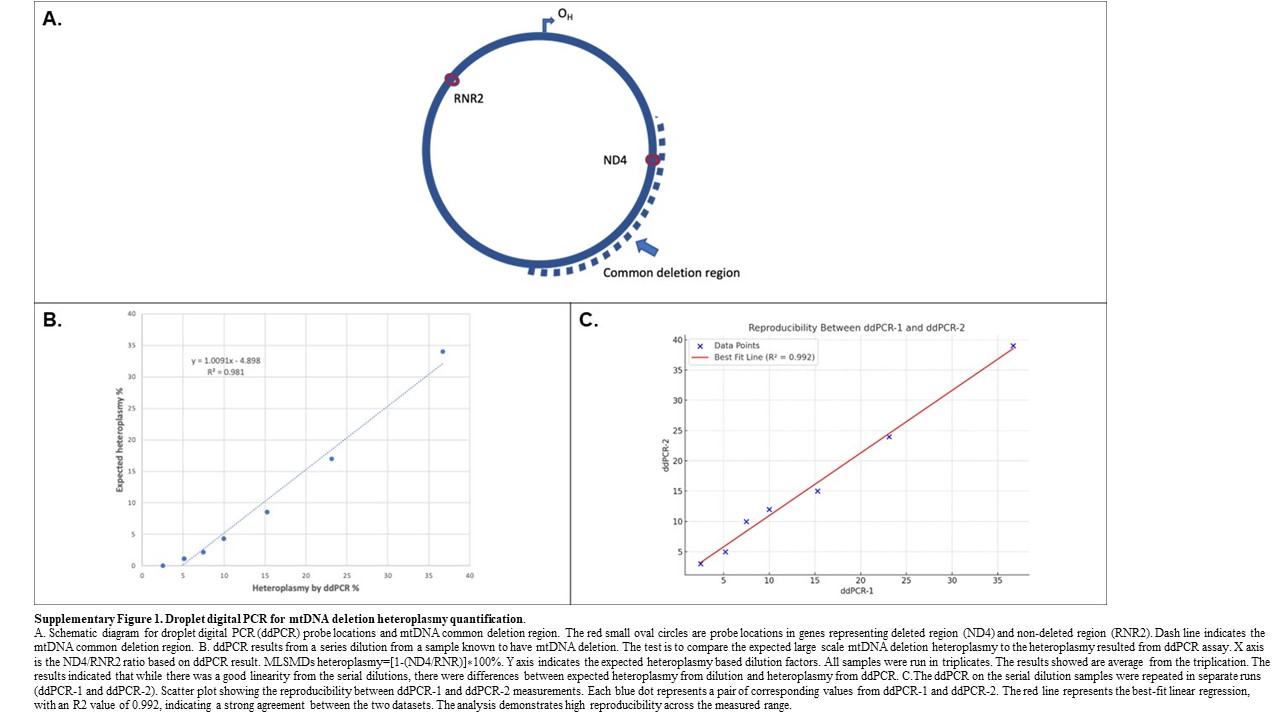

Supplement: Supplementary file 2 [file Image1.jpg]
